# Comparison of the Efficacy of Rosuvastatin versus Atorvastatin in Preventing Contrast Induced Nephropathy in Patient with Chronic Kidney Disease Undergoing Percutaneous Coronary Intervention

**DOI:** 10.1371/journal.pone.0111124

**Published:** 2014-10-30

**Authors:** Yong Liu, Yuan-hui Liu, Ning Tan, Ji-yan Chen, Ying-ling Zhou, Li-wen Li, Chong-yang Duan, Ping-Yan Chen, Jian-fang Luo, Hua-long Li, Wei-Guo

**Affiliations:** 1 Department of Cardiology, Guangdong Cardiovascular Institute, Guangdong General Hospital, Guangdong Academy of Medical Sciences, Guangzhou, Guangdong, China; 2 Southern medical university, Guangzhou, Guangdong, China; 3 Department of Biostatistics, School of Public Health and Tropical Medicine, Southern Medical University, Guangzhou, China; National Centre for Scientific Research “Demokritos”, Greece

## Abstract

**Objectives:**

We prospectively compared the preventive effects of rosuvastatin and atorvastatin on contrast-induced nephropathy (CIN) in patients with chronic kidney disease (CKD) undergoing percutaneous coronary intervention (PCI).

**Methods:**

We enrolled 1078 consecutive patients with CKD undergoing elective PCI. Patients in Group 1 (n = 273) received rosuvastatin (10 mg), and those in group 2 (n = 805) received atorvastatin (20 mg). The primary end-point was the development of CIN, defined as an absolute increase in serum creatinine ≥0.5 mg/dL, or an increase ≥25% from baseline within 48–72 h after contrast medium exposure.

**Results:**

CIN was observed in 58 (5.4%) patients. The incidence of CIN was similar in patients pretreated with either rosuvastatin or atorvastatin (5.9% vs. 5.2%, p = 0.684). The same results were also observed when using other definitions of CIN. Clinical and procedural characteristics did not show significant differences between the two groups (p>0.05). Additionally, there were no significant inter-group differences with respect to in-hospital mortality rates (0.4% vs. 1.5%, p = 0.141), or other in-hospital complications. Multivariate logistic regression analysis revealed that rosuvastatin and atorvastatin demonstrated similar efficacies for preventing CIN, after adjusting for potential confounding risk factors (odds ratio = 1.17, 95% confidence interval, 0.62–2.20, p = 0.623). A Kaplan–Meier survival analysis showed that patients taking either rosuvastatin or atorvastatin had similar incidences of all-cause mortality (9.4% vs. 7.1%, respectively; p = 0.290) and major adverse cardiovascular events (29.32% vs. 23.14%, respectively; p = 0.135) during follow-up.

**Conclusions:**

Rosuvastatin and atorvastatin have similar efficacies for preventing CIN in patients with CKD undergoing PCI.

## Introduction

Contrast-induced nephropathy (CIN) is an important and well-known complication in patients undergoing percutaneous coronary intervention (PCI). CIN also causes prolonged in-hospital stays and excess health care costs, and represents a powerful predictor of short and long term adverse outcomes [1], [2], [3]. CIN occurs even more frequently in patients with chronic kidney disease (CKD), with a reported incidence as high as 20–26.6% [3], [4]. However, other than periprocedural hydration with normal saline, limiting the amount of contrast medium (CM), and using iso- or low-osmolar CM, few strategies are effective for preventing CIN.

Statins belong to a drug class that has pleiotropic effects on the vasculature and improves endothelial function, probably by increasing nitric oxide synthetase bioavailability and decreasing oxidative stress [5], [6], [7]. These properties counteract specific pathophysiologic mechanisms that promote the development of CIN [2], [8]. In recent years, increasing evidence has supported the preventive effect of atorvastatin on CIN development in patients undergoing PCI [9], [10]. Additionally, two large randomized control trials (RCTs) demonstrated that rosuvastatin significantly reduced the risk of CIN and improved short term clinical outcomes [11], [12]. However, not all statins (especially, rosuvastatin and atorvastatin) are equivalent; they vary in several properties, including low-density lipoprotein (LDL) cholesterol lowering potency, lipophilicity, renoprotection, anti-inflammatory effects, and their effects on myocardial function [13], [14]. Whether these differences significantly influence their effect on preventing CIN remains unknown. Recently, Kaya et al. (ROSA-CIN trial) conducted a study including 198 ST-segment elevation myocardial infarction (STEMI) patients undergoing primary PCI to determine if rosuvastatin and atorvastatin had similar efficacies for preventing CIN [15]. However, the number of enrolled patients was too small to draw definite conclusions; additional large trials are required to confirm their similarity. Therefore, we performed a prospective study to compare the preventive effects of rosuvastatin and atorvastatin on CIN in patients with CKD undergoing PCI.

## Patients and Methods

### Patient population

We prospectively enrolled consecutive CKD patients undergoing PCI at Guangdong Cardiovascular Institute, Guangdong General Hospital, China, between March 2010 and September 2012. The inclusion criteria included: patients with an estimated glomerular filtration rate (eGFR) of 30–90 mL/min/1.73 m^2^ (CKD stages II and III), and patients pretreated with either atorvastatin (20 mg) or rosuvastatin (10 mg), at equivalent standard doses [16]. Statin pretreatment was defined as taking a statin 2–3 days before CM exposure and 2–3 days after the procedure. Patients were excluded if they had undergone chronic statin therapy (>14 days); had been treated with simvastatin or other statins; had a history of heart failure (defined as NYHA III/IV or Killip class II–IV), pregnancy, CM allergy, CM exposure during the previous 7 days; or had been treated with potentially nephroprotective (e.g., N-acetylcysteine or theophylline) or nephrotoxic (e.g., steroids, non-steroidal anti-inflammatory drugs, aminoglycosides, amphotericin B) drugs [17]. We also excluded patients with CKD stages 0, IV or V; hepatic insufficiency; or who had undergone renal transplantation or dialysis.

This study protocol was approved by the Guangdong General Hospital ethics committee and the study conformed to the Declaration of Helsinki. Written informed consent was obtained from all patients before the procedure.

### Biochemical investigations

Serum creatinine (SCr) levels were measured upon admission and within 48–72 h after CM exposure. Blood urea nitrogen (BUN), creatine kinase MB, fasting glucose, electrolytes, fasting lipid profiles, albumin, and other standard clinical parameters were measured in the morning before the procedure. The eGFR was evaluated using the 4-variable Modification of Diet in Renal Disease equation based on Chinese patients [18]. Left ventricular function was echocardiographically evaluated in each patient within a 24-h period before the PCI.

### PCI and medications

PCI was performed by experienced interventional cardiologists according to standard clinical practice using standard techniques. Nonionic, low-osmolar CM was used in all patients (either Iopamiron or Ultravist, both at 370 mg I/mL). Normal saline (0.9%) at a rate of 1 mL/kg/h (0.5 mL/kg/h if the patient’s left ventricular ejection fraction (LVEF) was <40%) was administered intravenously 3–12 h before and 6–12 h after CM exposure. Anti-platelet agents (aspirin/clopidogrel), β-adrenergic blocking agents, statins, diuretics, angiotensin-converting enzyme inhibitors, and inotropic drugs were used at the attending cardiologist’s discretion, according to clinical protocols derived from interventional guidelines.

### Clinical outcomes

Follow-up events were carefully monitored and recorded by trained nurses through office visits and telephone interviews conducted, at 1, 6, 12, and 24 months after cardiac catherization.

The primary end-point was CIN development, defined as an absolute increase in SCr ≥0.5 mg/dL or a relative increase ≥25% from baseline, within 48–72 h after CM exposure. Additional end points included: CIN, as defined by other criteria [17], and major in-hospital or long-term adverse clinical events (MACEs), including all-cause mortality, non-fatal myocardial infarction, target vessel revascularization, CIN requiring renal replacement therapy, and stroke.

The other CIN definitions included: an absolute increase in SCr of ≥0.5 mg/dL within 48–72 h (CIN2); an absolute increase in SCr of ≥0.3 mg/dL within 48 h (CIN3); a SCr increase of ≥50% (1.5 fold from baseline) within 48 h (CIN4); and CIN5 (CIN3 or CIN4) [17].

### Statistical analysis

SAS version 9.2 (SAS Institute, Cary, NC, USA) was used for all analyses. Continuous variables are described as means ± SD or medians, and categorical variables as absolute values (percentages). Comparisons of between-groups differences were performed using Student’s *t*-test or the Wilcoxon rank sum test (if not normally distributed) for continuous variables and a chi-square or Fisher’s exact test for categorical variables. Logistic regression analysis was performed using CIN as the dependent variable. Variables that were statistically significant according to a univariate analysis, were included in the final multivariate model to identify CIN predictors. Cumulative event curves for both groups of patients were created using the Kaplan-Meier survival method and compared using the log-rank test. All statistical tests were 2-tailed and statistical significance was inferred if P<0.05.

## Results

### Baseline characteristics between patients pretreated with atorvastatin and rosuvastatin

A total of 1078 consecutive CKD patients, pretreated with atorvastatin or rosuvastatin were analyzed (mean age, 65.2±10.1 years; mean eGFR, 69.8±14.0 mL/min/1.73 m^2^; mean Mehran score, 4.3±3.2). Clinical and procedural characteristics were not significantly different between the two groups. In particular, the proportions of patients with diabetes mellitus (DM, P = 0.091), age ≥75 years (P = 0.200), or anemia (P = 0.187) were similar in both groups. The baseline SCr (P = 0.495) and eGFR (P = 0. 704) levels were also similar between the two groups, as were the mean LVEF (rosuvastatin 59.96±11.18% vs. atorvastatin 59.05±11.77%, P = 0.291), CM volumes used (rosuvastatin 133.36±67.75 mL vs. atorvastatin 132.37±70.13 mL, P = 0.838), and Mehran risk scores (rosuvastatin 4.06±2.86 vs. atorvastatin 4.42±3.31, P = 0.095). (Table 1).

**Table 1:** Baseline clinical characteristics of study participants.

Variables	Rosuvastatin (n = 273)	Atorvastatin (n = 805)	P
**Demographics**			
Age, (y)	65.28±9.89	65.79±10.28	0.425
Age>75 y, (%)	36(13.2%)	126(15.7%)	0.443
Females (%)	57(20.9%)	187(23.2%)	0.423
Weight (kg)	65.58±10.18	65.17±10.24	0.409
SBP (mmHg)	133.07±21.64	131.01±20.44	0.158
DBP (mmHg)	76.64±11.44	75.31±11.23	0.093
Heart rate (bpm)	74.33±12.32	72.94±12.15	0.105
**Medical history, n (%)**			
Smokers	108(39.6%)	301(37.4%)	0.523
Hypertension	176(64.5%)	506(62.9%)	0.633
Diabetes	56(20.5%)	206(25.6%)	0.091
Dyslipidemia	41(15.0%)	112(13.9%)	0.651
Prior MI	31(11.4%)	100(12.4%)	0.641
Prior CABG	4(1.5%)	8(1.0%)	0.521
**Laboratory findings**			
Baseline SCr (µmol/L)	99.29±24.77	98.17±23.07	0.495
Baseline-eGFR DDEeGFR (mL/min/1.73 m^2^)	69.49±14.83	69.86±13.73	0.704
Log-NT-pro-BNP (pg/mL)	5.59±1.76	5.66±1.68	0.573
hs-CRP (mg/L)	12.02±21.96	10.10±19.77	0.281
LVEF, %	59.96±11.18	59.05±11.77	0.291
Total cholesterol (mmol/L)	4.23±1.08	4.29±1.94	0.660
Triglyceride (mmol/L)	1.44±0.89	1.79±8.17	0.329
LDL (mmol/L)	2.53±0.94	2.48±0.86	0.548
HbA1c, %	6.53±1.53	6.49±1.20	0.679
HG, g/L	132.21±14.77	132.57±16.54	0.733
Anemia, n (%)	86(31.5%)	289(35.9%)	0.187
Serum albumin, g/L	34.76±3.95	35.47±4.29	0.018
Uric acid, µmol/L	374.81±103.30	389.95±108.719	0.074
**Medication, n (%)**			
ACEI/ARB	242(88.6%)	729(90.6%)	0.361
β-bloker	237(86.8%)	720(89.4%)	0.235
Calcium channel blocker	70(25.6%)	163(20.2%)	0.061
Diuretics	27(9.9%)	101(12.5%)	0.241
**Procedural characteristic**			
Contrast volume (mL)	133.36±67.75	132.37±70.13	0.838
Contrast exposure time (min) (min)	73.37±43.97	71.96±47.34	0.669
Number of diseased vessels (n)	2.14±1.05	2.03±1.12	0.156
Number of stents (n)	1.68±1.20	1.60±1.19	0.387
Contrast volume/eGFR ratio	2.07±1.28	2.01±1.22	0.467
Mehran score	4.06±2.86	4.42±3.31	0.095

Abbreviations: SBP: systolic blood pressure; DBP: diastolic blood pressure. MI: myocardial infarction; CABG: coronary artery bypass grafting; SCr: serum creatinine; eGFR: estimated glomerular filtration rate; NT-pro-BNP: N-Terminal Pro-B-Type natriuretic peptide; hs-CRP: high sensitivity C reactive protein; LVEF: left ventricular ejection fraction; LDL: low density lipoprotein; HbA1c: hemoglobin A1c; HG: hemoglobin: ACEI/ARB: angiotensin-converting enzyme inhibitor/angiotensin receptor blocker; Mehran score: model to define contrast-induced nephropathy (CIN) by Mehran et al. Anemia was defined using World Health Organization criteria: baseline hematocrit value <39% for men and <36% for women.

### Preventive effect of statins on CIN and in hospital outcomes

**Figure 1:**
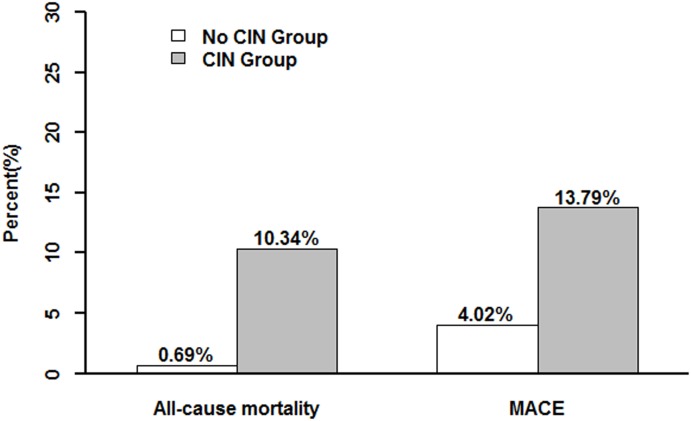
Multivariate logistic analysis associating contrast-induced nephropathy with various risk indicators.

Overall, CIN was observed in 58 patients (5.4%). Compared with patients without CIN, patients with CIN had a significantly higher rate of in-hospital mortality (10.34% vs. 0.69%, P<0.001), and other in hospital complications, such as the requirement for renal replacement therapy (3.4% vs. 0.4%, P = 0.002) and the use of intra-aortic balloon pump (IABP; 10.34% vs. 1.18%, P<0.001). (Figure 1).

The incidences of CIN were similar between patients pretreated with either rosuvastatin or atorvastatin (5.9% vs. 5.2%, P = 0.684); similar results were also obtained using the alternate CIN definitions. In addition, there were no significant differences between the two groups with regard to the rate of in-hospital mortality (0.4% vs. 1.5%, P = 0.141). However, patients treated with rosuvastatin had a lower incidence of in-hospital MACEs than those treated with atorvastatin (1.8% vs. 5.5%, P = 0.013) (Table2).

**Table 2:** In-hospital events in patients treated with rosuvastatin or atorvastatin.

Variables	Rosuvastatin (n = 273)	Atorvastatin (n = 805)	P
CIN1	16 (5.9%)	42 (5.2%)	0.684
CIN2	5 (1.8%)	13 (1.6%)	0.809
CIN3	10 (3.7%)	33 (4.1%)	0.750
CIN4	2 (0.7%)	10 (1.2%)	0.488
CIN5	10 (3.7%)	33 (4.1%)	0.750
Death	1 (0.4%)	12 (1.5%)	0.141
Renal replacement therapy	1 (0.4%)	5 (0.6%)	0.625
Hypotension	3 (1.1%)	16 (2.0%)	0.335
IABP	3 (1.1%)	15 (1.9%)	0.394
Acute heart failure	2 (0.7%)	11 (1.4%)	0.407
Cerebrovascular accident	0 (0.0%)	3 (0.4%)	0.312

Abbreviations: CIN: contrast induced nephropathy; IABP: intra-aortic ballon pump.

**Figure 2:**
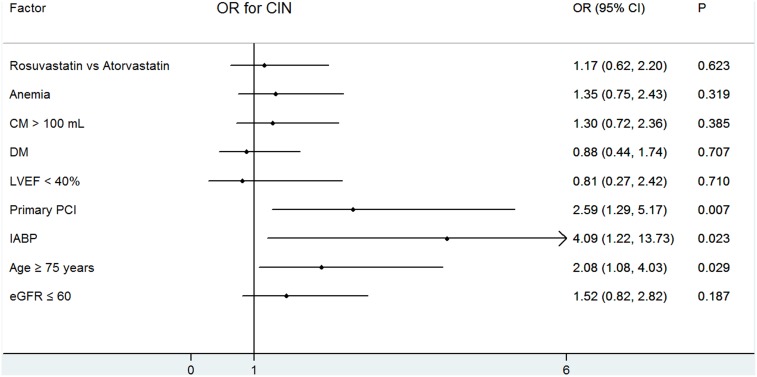
The prevalence of in-hospital all-cause mortality or major adverse cardiovascular events in patients with or without contrast-induced nephropathy.

Multivariate logistic regression analysis revealed that pretreatment with rosuvastatin had a similar effect as atorvastatin pretreatment regarding the development of CIN in patients undergoing PCI (odds ratio [OR] = 1.17, 95% confidence interval [CI], 0.62–2.20, P = 0.623), even after adjusting for potential confounding risk factors (age >75 years, eGFR ≤60 mL/min/1.73 m^2^, DM, anemia, CM >100 mL, IABP, LVEF<40%, primary PCI). Age >75 years (P = 0.029), IABP (P = 0.023), and primary PCI (P = 0.007) were other independent predictors of CIN in this population. (Figure 2).

### Clinical outcomes during follow-up

The median follow-up period was 2.51±0.86 years (inter quartile range, 1.80–3.27 years) and was continued for all patients who survived to discharge.

**Figure 3:**
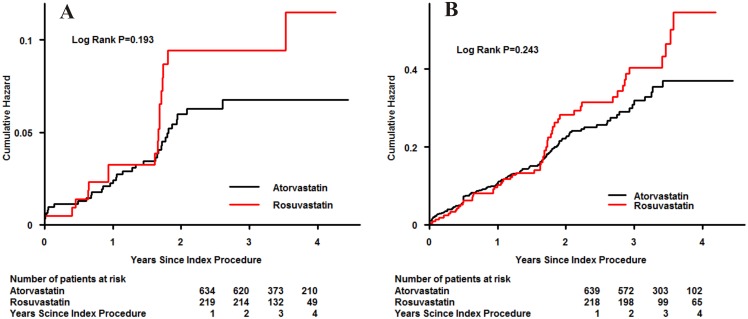
Cumulative rate of follow-up all-cause mortality (A) or major adverse cardiovascular events (B) in patients initially treated with rosuvastatin or atorvastatin.

**Figure 4:**
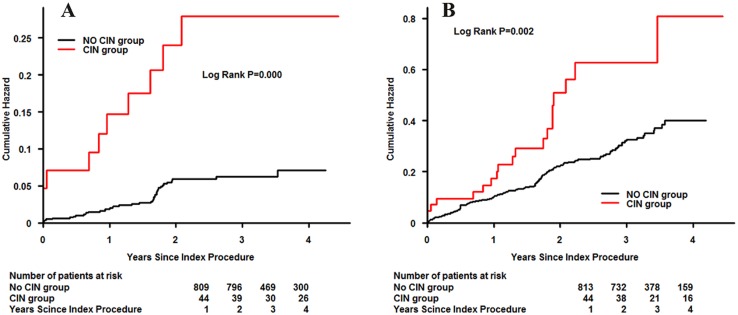
Cumulative rate of follow-up all-cause mortality (A) or major adverse cardiovascular events (B) in patients with or without contrast-induced nephropathy.

To determine the relationship between the accumulated risk of adverse events and rosuvastatin or atorvastatin pretreatment, a Kaplan–Meier survival analysis was performed. Patients pretreated either rosuvastatin or atorvastatin demonstrated a similar incidence of all-cause mortality (7.76% vs. 5.36%, P = 0.193) or MACEs (26.48% vs. 21.28%, P = 0.243), as illustrated in Figure 3. In addition, patients who developed CIN had a higher rate of all-cause mortality than those who did not (cumulative rate of mortality, 22.73% vs. 5.07%, P<0.001). A similar result was found for MACEs. (43.18% vs. 21.50%, P = 0.002). (Figure 4).

## Discussion

The present study may be the first to demonstrate that pretreatments with either rosuvastatin or atorvastatin have similar efficacies for preventing CIN in patients with CKD undergoing PCI.

The prevention of CIN is an important concern because it affects patient morbidity and mortality, especially in CKD patients [3], [4]. In the current study, we found that the incidence of CIN was 5.4%, in agreement with previous studies [3]. Similar to previous studies, we found that patients developing CIN had a higher risk of poor in-hospital and long-term clinical outcomes. Because, few strategies have been demonstrated to be effective for preventing CIN [17]. The development of new strategies to decrease CIN occurrence, especially for high-risk CKD patients is urgently needed. This has led to an increased interest in the preventive effects of statins (especially, atorvastatin and rosuvastatin) on CIN development in patients undergoing PCI.

However, conflicting results have been published. Kandula et al [19] reported an observational study (239 patients with statins, 114 without statins), that showed statin treatment was not associated with CIN prevention, after adjusting for the propensity of receiving statins (OR = 1.6, 95% CI: 0.86–3.22, P = 0.12). In contrast, another study based on a database of 29,409 patients undergoing emergent and non-emergent PCI [20], reported that patients using statins had a lower risk of CIN than did those not using statins (4.4% vs. 5.9%, P<0.001). Similar results were demonstrated by Hoshi et al [21]. Other than these observational studies, many RCTs have been conducted to address this topic. Toso et al [22] performed a prospective RCT, including 304 patients, to investigate the efficacy of short-term high dose atorvastatin on preventing CIN development in patients with CKD undergoing PCI. The results showed that short-term high doses of atorvastatin, administered periprocedurally, did not decrease CIN occurrence in patients with pre-existing CKD. However, another group [10] enrolled 410 patients with CKD in an RCT and demonstrated that a single high dose of atorvastatin administered within a 24 h period before CM exposure, was effective at reducing the CIN rate. Similar findings have been reported from subsequent RCTs [9], [21], [23]. A previous meta-analysis of 7 RCTs, with a total of 1399 patients (693 patients receiving high-dose statins, 706 receiving low-dose or no statins) revealed that atorvastatin was beneficial for preventing of CIN [24], which is in agreement with our recent meta-analysis [25].

Two large RCTs recently demonstrated that rosuvastatin pretreatment, upon admission, could reduce CIN occurrence in patients undergoing PCI. Leoncini et al [11] reported that acute coronary syndrome patients, without ST-segment elevation, who were treated with rosuvastatin (40 mg on-admission, followed by 20 mg/day) experienced less CIN than patients not receiving rosuvastatin. Similarly, in patients with type 2 DM and CKD, another group showed that rosuvastatin significantly reduced the risk of CIN after CM exposure [12]. Accordingly, although guideline committees have not recommended this CIN-prevention strategy, researchers are increasingly considering statins as an effective drug for preventing CIN, based on the existing evidence.

Although the mechanism of statins in CIN prevention remains unknown, the following mechanisms may play important roles. In addition to their intended impact on blood cholesterol levels, statins are also known to have pleiotropic effects. Previous studies showed that statins treatment could prevent renal tubular cell apoptosis and increase survival signaling pathways [10]. However, the direct toxic effects of CM on renal cells, leading cell necrosis or apoptosis, are thought to contribute to the CIN pathogenesis. Preventing CM-induced renal cell apoptosis seems to play an important role in the statins’ effects on CIN [10]. In addition, endothelial dysfunction, another major contributor to CIN progression, is caused by a nitric oxide (NO) and endothelin-1 imbalance, after CM exposure. Statins may help correct this imbalance by increasing NO production and reducing endothelin-1 synthesis [26]. Furthermore, C-reactive protein (CRP), as a marker of systemic inflammation, is also associated with CIN, and patients with high periprocedural CRP levels are at high risk for developing CIN [9], [27], [28]. Recent studies have demonstrated that the preventive effect of statins on CIN development parallels a significant decrease in post-procedural CRP levels [12]. Thus, statins may reduce inflammation by inhibiting pro-inflammatory mediator synthesis [29], and may have a reno-protective effect during CM exposure by attenuating inflammatory responses.

Different statins (e.g., atorvastatin and rosuvastatin) vary in their LDL-lowering potency, lipophilicity, reno-protection, and anti-inflammatory effects [13], [14]. However, whether the difference (hydrophilic and lipophilic) between statins influences their ability to reduce CIN risk is unclear. Rosuvastatin, a hydrophilic statin, has acute pleiotropic effects, and has been demonstrated to reduce LDL more aggressively, without increasing complications, and improve patient prognosis better than the other statins [30]; it also, exerts a beneficial reno-protective effect in patients with renal dysfunction [31]. Additionally, rosuvastatin has a longer plasma half-life and stronger anti-inflammatory effects than atorvastatin [32], [33]. Because patients with CKD have significantly higher mean CRP levels [34], rosuvastatin may be more effective in these patients. Furthermore, Thiago et al demonstrated that rosuvastatin performed better than atorvastatin or simvastatin, in an experimental murine model of cigarette smoke-induced acute lung inflammation, because of better attenuation of both inflammation and oxidative stress parameters [35]. A recent meta-analysis reported that rosuvastatin might also increase apolipoprotein A-I levels at all doses more than atorvastatin [36]; apolipoprotein A-I can stabilize lipoprotein structure and has anti-inflammatory and antioxidant properties [37]. Based on these difference between rosuvastatin and atorvastatin, we hypothesized that rosuvastatin would differ from atorvastatin with respect to their abilities to prevent CIN.

To date, large studies investigating the CIN-risk reduction differences between rosuvastatin and atorvastatin have not been reported. One recent study, including 192 patients (94 taking rosuvastatin, 98 taking atorvastatin), compared the effects of different statins on CIN in STEMI patients treated with primary PCI; both statins had similar efficacies for preventing CIN. The study also suggested that the incidence of Killip class ≥2 patients ranged from 91.8–94.7% [15]. Therefore, increased of SCr in those patients may be the result of hemodynamic compromise due to acute impairment of cardiac pump function after extended myocardial infarction, rather than the direct effect of CM exposure [38]. However, in our study, the patients had relatively stable hemodynamic status because patients with a history of heart failure (NYHA ≥ III and Killip ≥ II) were excluded. Thus, CM administration may play a major role and the reduced risk of CIN may be a true reflection of the statins’ effects. In our study, patients receiving rosuvastatin displayed higher levels of hs-CRP than did those treated with atorvastatin, suggesting that these patients would be more likely to develop CIN, based on the previous evidence [27], [28]. However, our findings demonstrated that the incidence of CIN in rosuvastatin-treated patients was similar to that in atorvastatin- treated patients; the patients were relatively well balanced with respect to their baseline clinical and angiography characteristics. Although we did not demonstrate that rosuvastatin was superior to atorvastatin for preventing CIN, the results may not be surprising considering that different factors are involved in CIN development and that different patho-physiological mechanisms coexist.

The present study also demonstrated the patients pretreated with rosuvastatin or atorvastatin had similar risks of all-cause mortality and MACEs. In addition, we demonstrated that age >75 years, IABP use, and primary PCI were independent risk factors of CIN, but not an eGFR ≤60 mL/min/1.73 m^2^. However, Ando et al have demonstrated that eGFR as a continuous variable was a risk factor for CIN in STEMI patients treated with primary PCI [39]. This might be related to the different patient populations included in the two studies.

### Limitations

There are several limitations to this study. First, this was a prospective, observational study conducted at a single center. Therefore, causality cannot be ascribed. Second, our study population was limited to CKD (stage II and III) patients, so the results may not extend to patients with other stage of CKD or those without CKD. Third, due to variations in the timing of measurements, we may have missed the post-procedural SCr peak. Furthermore, we did not use cystatin C which is a more sensitive biomarker and increases faster than SCr after CIN. Thus, the true incidence of CIN may have been underestimated. Fourth, SCr levels were not systematically measured during the follow-up period. Fifth, in consideration of previous studies revealed that high-dose atorvastatin (40 or 80 mg) pretreatment was more effective than low-dose (20 mg) or no statin therapy [24], we did not investigate the protective efficacies of different doses in our study.

## Conclusions

Our study demonstrated that rosuvastatin pretreatment exerts an effect similar to atorvastatin in preventing CIN in high risk patients with CKD undergoing PCI. Thus, future head to head studies are required to compare hydrophilic and lipophilic statins to determine if they reduce CIN risks differently.
